# Virtual reality, real emotions: a novel analogue for the assessment of risk factors of post-traumatic stress disorder

**DOI:** 10.3389/fpsyg.2015.00681

**Published:** 2015-05-22

**Authors:** Pauline Dibbets, Michel A. Schulte-Ostermann

**Affiliations:** ^1^Clinical Psychological Science, Faculty of Psychology and Neuroscience, Maastricht University, Maastricht, Netherlands; ^2^Institut für Sexualmedizin und Forensische Psychiatrie und Psychotherapie, Universitätsklinikum Schleswig-Holstein, Kiel, Germany

**Keywords:** virtual reality, trauma induction, post-traumatic stress disorder, risk factors, imagery ability

## Abstract

Most people are exposed to a violent or life-threatening situation during their lives, but only a minority develops post-traumatic stress disorder (PTSD). Experimental studies are necessary to assess risk factors, such as imagery ability, for the development of PTSD. Up to now the trauma film paradigm (TFP) has functioned as an analogue for PTSD. This paradigm is known to induce involuntary intrusions, a core symptom of PTSD. Though useful, the film paradigm has a drawback, the participant remains an “outsider” and does not immerse in the film scenes. The aim of the present study was to develop a fitting virtual reality (VR) analogue for PTSD and to assess risk factors for the development of PTSD-symptoms, such as intrusions. To this end a novel VR paradigm was compared to the traditional TFP. Both the VR and TFP elicited a negative mood and induction-related intrusions. More immersion was observed in the VR paradigm compared to the TFP. The results of the risk factors were mixed; more imagery ability coincided with a higher intrusion frequency, but also with less distressing intrusions. The results, implications and suggestions for future research are discussed.

## Introduction

Almost everybody has experienced, or will experience, at least one event in life that is so intense, that it is judged as traumatic. In the initial aftermath of such traumatic experience intrusive memories are very common. These involuntary re-experiences can occur in several forms such as images, noises, smells, flashbacks, or nightmares. Intrusive memories cause significant distress and interfere with daily functioning. Situations or activities that might trigger reminders of the traumatic event are avoided, isolating a patient from his or her social environment. If these intrusive memories persist over a long time, they may lead to post-traumatic stress disorder (PTSD, e.g., Ehlers and Steil, 1995). About 21% of the victims that is exposed to a violent life threatening assault shows persistent symptoms and develops PTSD (Breslau et al., 1998). This implies that 79% does not develop PTSD. This raises the question, why do some persons develop PTSD and others not?

Over the past decades, several correlational studies have addressed potential risk and vulnerability factors for the development and maintenance of PTSD (Breslau et al., 1998; Brewin et al., 2000). Though informative, these studies can only retrospectively assess these factors. Such methodology has important limitations as PTSD patients find it hard to accurately describe past emotional states or events. Furthermore, patients vary on the type and severity of the trauma experienced, the duration of the traumatic experience and the time between assessment and trauma. Therefore, experimental studies are more suitable to test causal theories about the development and risk factors of PTSD. However, it is clearly unethical to expose a person to a real traumatic event in the laboratory. Therefore, researchers have often drawn on analogue designs to gain more insight in PTSD. Up to now, the most common analogue design uses stress-evoking film fragments. The traumatic content of these fragments can either be realistic, such as real traffic accidents, or fictitious, such as a rape scene derived from a film (Weidmann et al., 2009). This paradigm, referred to as trauma film paradigm (TFP, Holmes and Bourne, 2008), enables experimental manipulations and assessment of factors that affect the development and maintenance of PTSD.

In response to a stressful film, individuals have been found to demonstrate short-term PTSD-like symptoms, such as negative mood, distress, dissociation, and intrusive memories for film content (Holmes and Bourne, 2008). Though the TFP does evoke PTSD-like symptoms, the paradigm has its drawbacks. First of all, there is still a debate as to whether PTSD-symptoms can be evoked via television, especially if there is no direct relationship between the viewer and the victims (Pfefferbaum et al., 2002). Second, the number of reported intrusions evoked by a stressful film is small; the average number of intrusions in the 3 days following the film varies between 1.6 (traffic accidents; Halligan et al., 2002) and 5.92 (rape scene; Weidmann et al., 2009). This is not considered to be problematic as not the frequency, but the distress caused by the intrusions, their “here and now” quality, and their lack of an embedded context are thought to predict PTSD severity (Michael et al., 2005). However, film-evoked intrusions are rated as moderately vivid (Weidmann et al., 2009), and little distress is reported (Halligan et al., 2002).

In a recent study we used one of the most stressful films (Salò o le 120 giornate di Sodoma), resulting in an average of 4.4 intrusions in the week following the film (Dibbets and Arntz, in press). However, the intrusive memories caused only mild arousal, were moderately vivid, and participants perceived them as highly controllable. Post-experimental interviews revealed that participants were constantly aware that it was “only” a film with actors; resulting in problems imagining being a close witness at the scene and leaving a high level of control.

New technologies provide us with novel clinical tools. Recently, virtual reality (VR) was implemented to prevent and treat PTSD (see for an overview of VR exposure therapy, VRET, Rizzo et al., 2011; Gonçalves et al., 2012). Using VR, a person is no longer a passive observer, but can immerse and interact in a virtual 3D environment. VR research has indicated that especially this feeling of “presence” is related to the level of emotions, such as anxiety (Riva et al., 2007), and that, compared to a 3D-film, VR experiences resulted in more immersion and more intensive emotions (Visch et al., 2010). VR is already applied in exposure therapy, VRET, including combat-related PTSD (Rizzo et al., 2011), and in stress resilience training for military (Rizzo et al., 2011) or emergency medicine personnel (Andreatta et al., 2010). Therefore, VRET seems to be as effective as *in vivo* exposure in reducing PTSD symptoms. This is highly relevant as it is not always possible to exposure patients to trauma-related material and some patients find it difficult to immerse themselves in a traumatic scene (Gonçalves et al., 2012). Recently, immersive VR has been applied in an experimental setting to study bystander interventions in interpersonal violent incidents (Slater et al., 2013). This study indicated that individuals can react realistically in VR and emotions were reported that were in line with the violent scene displayed (i.e., feelings of helplessness, fear or anger). However, to our knowledge, VR has never been applied as an analogue for trauma experiences, evoking PTSD-symptoms. Given the fact that VR results in more immersion and more intense emotions, VR might evoke intrusions that are more comparable to the intrusions that PTSD patients experience, making the results of analogue trauma experiments more relevant for clinical settings. Therefore, the first aim of the present study was to develop a VR analogue for PTSD-symptoms and to compare this analogue with a TFP.

The development of such a new VR paradigm can help to identify pre-existing emotional and personality vulnerability factors that might account for individual differences for the development and maintenance of PTSD. Factors that have been repeatedly associated with PTSD are trait anxiety (Halligan et al., 2003) and depression (Halligan et al., 2003). Similar correlations are observed in non-clinical persons; higher trait anxiety and depression are associated with more intrusions after watching a distressing film (Laposa and Alden, 2008).

One risk factor that has received considerably less attention is the ability to imagine objects or events. This is strange as intrusions, mainly concerning visual images, are at the core of PTSD. It is conceivable that PTSD patients, who suffer from intrusions, may experience their trauma in a vivid manner because of pre-existing imagination skills (Brett and Ostroff, 1985). Only a few studies have assessed the relation between imagery ability and PTSD symptoms. These clinical studies indicated that better imagery capacities coincided with more PTSD symptomatology (Stutman and Bliss, 1985) and more flashbacks and nightmares (Bryant and Harvey, 1996).

Though these studies indicate that imagery abilities, PTSD, and re-experiences are related, they only provide indirect, retrospective information. To our knowledge, only one study has examined the link between mental imagery ability and posttraumatic intrusions (Morina et al., 2013). In this study the level of mental imagery correlated positively with the amount, vividness and emotional distress due to intrusive images. The present study wants to extend the literature on risk factors, among which mental imagery, for PTSD-symptoms.

Based on previous studies we expected that (1) the VR paradigm would result in more feelings of immersion during the trauma induction compared to the traditional TFP; (2) VR trauma induction would result in higher levels of arousal and vividness and less perceived control of the intrusions compared to the TFP condition; (3) higher levels of the risk factors trait anxiety, depression and imagery ability would predict more frequent and intense intrusions.

The present study is a first step toward a novel analogue for PTSD in which participants are actually part of the aversive, violent scene (see also Slater et al., 2013). This is highly important as up to now film scenes were used resulting in low feelings of immersions and intrusions that were highly-controllable and moderately vivid and distressing. At the same time risk factors, among which imagery ability, for PTSD were examined. The results can help to gain further insight in the mechanisms and factors for the development and maintenance of PTSD.

## Materials and Methods

### Participants

A total of 43 participants (11 males, 32 females) was recruited from Maastricht University, Psychology Department, via advertisements on pin boards and social media. Participants were mainly students from Maastricht University (*n* = 41), one person worked as a web designer, and one person recently graduated. Participation was rewarded with either study credits or with a voucher of 15 euros.

### Ethical Concerns

Ethical permission was granted by the Ethical Committee of Maastricht University (ECP-131). As the present study was the first to use VR to induce a trauma, a number of safety strategies was adopted. First, knowing the VR results in more immersion than a (3D) film, the intensity of the VR scene was lower than of the film scene. Second, only persons without prior history of (witnessing) physical and/or sexual abuse were invited to participate. Third, a healthcare psychologist was part of the project team and was available for consultation by the participants.

The participants were debriefed after the experiment by providing written information about the experimental set up and main aims of the study. Contact information of the healthcare psychologist was provided at the bottom of the debriefing.

### Materials

#### Questionnaires

***Jellinek-PTSD screening questionnaire***

The JPSQ is a short self-report questionnaire and serves as a first screening instrument to identify participants which might suffer from PTSD (van Dam et al., 2013). The instrument consists of four questions that can be answered with either yes or no. The score is the total sum of positive answers (range 0–4). The JPSQ has shown to have high sensitivity (0.87) and specificity (0.75) (van Dam et al., 2013).

***Dutch version of the beck depression inventory II (BDI-II-NL)***

The Dutch version of the BDI-II (Beck et al., 1996) was used to assess the presence of depressive symptoms in the past 2 weeks (BDI-II-NL, Van der Does, 2002). The BDI-II-NL consists of 21 statements that can be answered on a four point scale with higher scores representing more depression (e.g., 0 = I don’t cry any more than I used to; 1 = I cry more than I used to; 2 = I cry over every little thing; 3 = I feel like crying, but I can’t). The total score can range between 0 and 63. The BDI-II-NL has been shown to have a high internal consistency (0.92 patients and 0.88 healthy controls) (Van der Does, 2002). In the present study, Cronbach’s α was 0.79.

***Questionnaire upon mental imagery***

To assess imagery ability, the QMI was applied (Sheehan, 1967). This questionnaire measures imagery ability in the following seven domains: visual, olfactory, cutaneous, organic, auditory, gustatory and kinesthetic. Each domain contains five items, resulting in a total of 35 items. Higher ratings indicate lower imagery ability and vice versa. This measurement has been reported to have a test–retest reliability coefficient of up to 0.91 (Evans and Kamemoto, 1973) and Cronbach’s α values between 0.77 and 0.94 (Westcott and Rosenstock, 1976; van de Ven and Merckelbach, 2003). In the present study, Cronbach’s α was 0.91 for the total score and ranged between 0.60 (visual) and 0.81 (taste) for the subscales.

***State-trait anxiety inventory DY***

The STAI-DY was used to assess trait anxiety prior to the experimental manipulation (Van der Ploeg, 1982). The STAI-DY consists of 20 items, which can be scored on a four point scale (e.g., I feel secure; 1 = not at all; 2 = somewhat; 3 = moderately; 4 = very much), with higher total scores indicating higher levels of trait anxiety. Cronbach’s α rangers from 0.89 to 0.93 and test–retest reliability ranged between 0.84 and 0.88 (Van der Ploeg, 1982). For the present study Cronbach’s α was 0.83.

***Mood ratings***

Mood was assessed by a short Mood Ratings questionnaire consisting of six items, asking for the experienced level of anxiety, disgust, control, gloom, anger and tension. Each item could be scored on a Likert-scale, ranging from 0 (not at all) to 10 (extreme). Higher scores indicate a higher negative mood. Cronbach’s α ranged from 0.63 (prior) to 0.80 (post).

***Independent television company-sense of presence inventory***

The ITC-SOPI is a self-report questionnaire that measures feelings of being (psychologically) present in a scene and can be used for various media devices (Lessiter et al., 2001). The questionnaire consists of 44 items, referring to physiological and psychological states during and after the experience of a presented scene. All items are rated on a scale ranging from one to five and can be subdivided into the following four factors: spatial presence, engagement, ecological validity/naturalness and negative effects. The first factor (spatial presence) captures the feeling of being physically present at the scene, whereas the second factor (engagement) relates to psychological involvement in the presented scene(s). Ecological validity/naturalness refers to the perceived reality of the material and negative effects are defined as negative physiological reactions to the apparatus (e.g., dizziness, headache). The ITC-SOPI has been reported to have a high internal reliability with Cronbach’s α ranging from 0.76 to 0.94, as well as a good content validity, differentiating between various media devices (Lessiter et al., 2001). In the present study, Cronbach’s α ranged between 0.52 (engagement) and 0.93 (spatial presence).

#### Virtual Reality Apparatus

The VR lab of the University of Maastricht is a room of six by four meters equipped with 16 speakers and cameras that are part of the highly accurate Phasespace tracking system^1^. Participants can move freely wearing a backpack with wireless receivers and a head mounted display (HMD, Nvis ST-50) that provides a 3D stereoscopic view. The virtual environment automatically adjusts to the participant’s head motions and orientation. The task was programmed in Python (via Vizard^2^), graphical content was made with Blender 3D^3^ and 3Ds Max (Motionbuilder^4^). Using this experimental set-up, the participant is free to walk around, can locate sounds and is able to explore the 3D environment. This helps to optimize immersion in the VR scenes.

#### Film

A scene, extracted from the movie “The Killer Inside Me” (Winterbottom, 2010), was used as the traumatic material for the TFP condition. The traumatic scene depicted a woman, who is severely physically assaulted by her lover at home, lasting for 2.30 min in total. This scenario was chosen, as it offered the possibility to create a similar scenario in the VR environment (VRE), in order to establish a fair comparison between the two paradigms. Additionally, other non-traumatic scenes of the same movie were presented for a coherent story line in order to enhance engagement of the participant (e.g., abuser driving his car to the victim’s house).

#### Virtual Reality Scene

**FIGURE 1:**
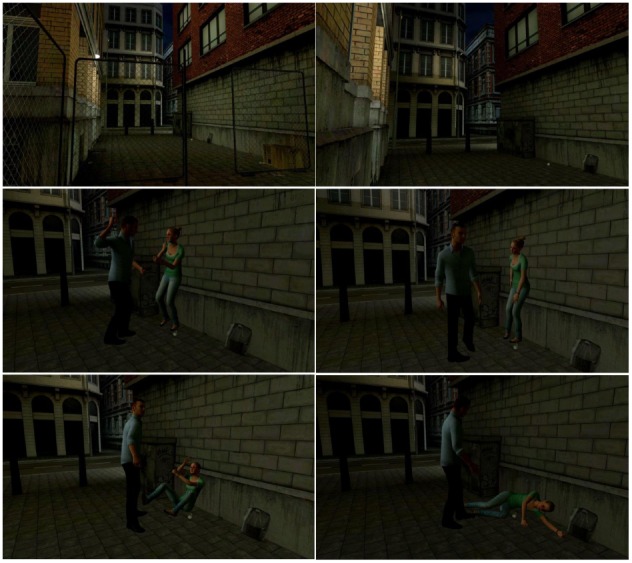
**Screenshots from the virtual reality scene**.

The VR scene was created by a digital artist (see for a portfolio^5^) and depicted a distressing scene between a woman and a man (see Figure 1). The general content of the VRE was similar to the corresponding movie scenes; a woman was severely physically assaulted by her lover. The scene took place in a dark and small alley, including background noises of a city and also lasted for 2:30 min. At the beginning of the scene, the couple entered the alley having an argument. The participant was located at the other end of the alley, from a first person’s point of view. After a few seconds, the man started to attack the woman with increasing intensity. The scene ended with the woman lying motionless on the ground and the man leaving the alley.

Using a violent, interactive VR scene is in line with the study of Slater et al. (2013). The interactive scene in this study elicited feelings of anxiety and fear and the desire to stop the aggressor. The VR scene was less intense compared to the film in that no close-ups of the woman’s face were presented and her injuries were less severe (e.g., less blood). In order to make the scene appear as realistic as possible, the movements of the characters were based on real-life movements, recorded by motion-capture techniques and afterward manually adjusted. The dialog of the couple and their voices were based on recordings of two actors from the Maastricht actor academy.

For both the film and VR scene, the aim was to increase negative emotions such as fear and anger. Both scenes depicted physical abuse with the victim left severely injured/dead on the floor. Using such intense scenarios increases chances of inducting PTSD-like symptoms (see also Brewin et al., 2000).

#### Intrusion Diary

For 7 days after the trauma induction, the participant recorded induction-related intrusions on a paper tabular diary (cf. Brewin and Saunders, 2001; Holmes et al., 2004; Hagenaars and Arntz, 2012). They noted the content of each intrusion (what was the intrusion about?), the situation that triggered the intrusion, the valence of the emotion accompanying the intrusion, and the level of distress, vividness, control and spontaneity on a scale from 0 to 100 (with 0 representing low levels and 100 high levels). Furthermore, the participant noted whether the intrusion was a thought, an image or a combination. The usage of the diary was explained verbally and written information was provided on the top of each page of the diary. For the present study, an intrusion was defined as an unintended “spontaneously occurring” memory containing at least a visual component.

### Procedure

#### Session 1: Trauma Induction

Participants without prior history of (witnessing) physical and/or sexual abuse were invited to take part. Participants received an email with the JPSQ. Only participants with a 0 score on the JPSQ (no PTSD symptoms) were invited to the lab. Participants were tested individually. After reading information about the general experimental set-up and signing an informed consent the participants received the BDI-II-NL; a score of 20 or higher resulted in termination of the experiment (see Morina et al., 2013). Subsequently, the QMI, Mood ratings, and STAI-DY were administered using a laptop. Participants were randomly assigned to either the TFP condition or the VR condition with the restriction of an equal gender distribution across conditions.

***TFP condition***

In the TFP condition the participants were seated in a comfortable armchair and audio was provided via a headphone. The participants were instructed to immerse completely into the depicted film scenes. Then the experimenter dimmed the light and left the room. By pressing spacebar, the participants started the film compilation. After watching the film the participant left the room and notified the experimenter who was waiting outside.

***VR condition***

The VR condition started with an explanation of the equipment. After gearing up, the participants were allowed to walk around in a virtual office to get acquainted with the equipment. Equal to the TFP condition, the participants were asked to immerse completely into the depicted scenes. On sign of the participants the aversive scene started. The experimenter guided the participants to the starting spot and remained in the laboratory to ensure that the participants remained within the physical space of the VR lab. The participants were instructed to approach the fighting couple as close as comfortable. At the end of the scene, participants returned to the starting point, the VR scene was ended by the experimenter and the VR equipment was removed.

After the trauma induction Mood ratings were assessed a second time and the ITC-SOPI was administered. Note that no relaxation session or debriefing of the experience took place. The reason for this was twofold; first to create an experience that closely resembles a real traumatic experience and, second, to optimize the occurrence of intrusions. Subsequently, the use of the Intrusion diary was explained and the telephone number of a healthcare psychologist was provided in case the participants needed counseling due to the experimental session.

#### Session 2: Follow-up

After exactly 7 days participants returned to the lab and handed over their Intrusion diary. The diary was shortly discussed and, if necessary, adjusted (e.g., omissions or ambiguities). The participants received course credit or a voucher and were debriefed.

### Statistical Analyses

A total of four participants was excluded, two participants had non-zero scores on the JPSQ and one person had a score of 21 on the BDI-II-NL. The last participant (TFP condition) was excluded from the data analyses as the number of intrusions (*n* = 43) deviated more than 3 SDs from the TFP average (*M*: 5.65, SD: 9.48), leaving a total sample of 39 (VR: *n* = 20, TFP: *n* = 19). The scores of the Mood rating, JPSQ, QMI and STAI-DY, BDI-II-NL and the frequency and characteristics of the intrusions served as dependent variables; experimental condition (TFP and VR) served as independent variable. Only intrusions that contained at least a visual image were included in the data analyses; for intrusion characteristics only participants with at least one (visual) intrusion were included. Data were analyzed using parametric tests, regression analyses (standardized β-values and adjusted *R*^2^), GLM repeated measures and ANOVAs. Possible differences between conditions regarding gender were analyzed non-parametrically. The rejection criterion was set at *p* < 0.05 throughout.

## Results

### Demographic Information

Table 1 displays the demographic information and mean scores on the questionnaires used. Prior to the trauma induction no differences between the TFP and VR condition were observed regarding gender distribution, χ^2^(1) = 0.065, *p* = 0.80, age, BDI-II-NL, STAI-DY or QMI scores, ANOVA, *F*s(1, 37) < 1.81, *p*s > 0.18, ${\text{η}}_{p}^{2}$ < 0.047.

**TABLE 1:** **Demographic information and means ( +SD) on the questionnaires**.

****	**TFP *N* = 19**	**VR *N* = 20**
Male/female	5/14	6/14
Age	22.16 (3.15)	23.45 (5.31)
BDI-II-NL	3.74 (3.07)	3.00 (4.05)
STAI-DY	35.58 (5.84)	33.00 (6.13)
QMI	83.89 (17.55)	79.20 (18.15)
Mood ratings 1	1.50 (0.90)	1.54 (1.04)
Mood ratings 2	3.94 (1.80)	3.13 (1.32)

### Trauma Induction

A repeated measures with Mood Ratings (before and after the induction) as within-subject factor and condition as between-subjects factor was carried out to assess the impact of the trauma induction on the (negative) mood. This analysis revealed a main effect of time, *F*(1, 37) = 57.45, *p* < 0.001, ${\text{η}}_{p}^{2}$ = 0.61, indicating an increase in negative mood. No effect of condition was observed, *F*(1, 37) = 1.39, *p* = 0.25, ${\text{η}}_{p}^{2}$ = 0.036, and no time × condition interaction was found, *F*(1, 37) = 2.54, *p* = 0.12, ${\text{η}}_{p}^{2}$ = 0.064.

### Immersion

Table 2 summarizes the means of the ITC-SOPI scales per condition. An ANOVA on the subscales of the ITC-SOPI showed differences between the conditions for the sense of spatial presence, *F*(1, 37) = 19.21, *p* < 0.001, ${\text{η}}_{p}^{2}$ = 0.34, a significant effect for psychological engagement, *F*(1, 37) = 4.96, *p* < 0.05, ${\text{η}}_{p}^{2}$ = 0.032, with VR resulting in more sense of spatial presence and engagement; no differences were observed for naturalness or negative effects, *F*s < 1.

**TABLE 2:** **Mean scores ( +SD) on the ITC-SOPI and Intrusion diary**.

****	**TFP *N* = 19**	**VR *N* = 20**
**ITC-SOPI**		
Spatial presence	2.36 (0.63)	3.19 (0.55)
Engagement	2.76 (0.36)	3.02 (0.36)
Naturalness	3.35 (0.68)	3.33 (0.76)
Negative effects	2.25 (0.81)	2.25 (0.57)
**Intrusion diary**		
Frequency	3.68 (3.65)	2.75 (1.83)
Distress	21.23 (22.81)	7.74 (13.29)
Vividness	36.29 (25.20)	38.80 (28.63)
Spontaneity	63.56 (27.83)	62.81 (29.87)
Control	72.43 (26.27)	87.63 (18.70)

### Intrusions

The number and characteristics of the intrusions are listed in Table 2. An ANOVA indicated that the total number of intrusions did not differ between the two conditions, *F*(1, 37) = 1.04, *p* = 0.32, ${\text{η}}_{p}^{2}$ = 0.027.

Only participants with at least one intrusion were included in the data analyses of the intrusion characteristics. The number of participants with intrusions in the TFP (*n* = 19) and VR condition (*n* = 18) did not differ, χ^2^(1) = 2.00, *p* = 0.16. Concerning the characteristics of the intrusions, no differences were observed concerning the vividness and spontaneity of the intrusions, *F*s < 1. However, the TFP resulted in more distress of the intrusions, *F*s(1, 33) < 4.34, *p*s < 0.05, ${\text{η}}_{p}^{2}$ = 0.12, compared to the VR condition. A marginally significant effect was observed for perceived control, *F*(1, 33) = 3.75, *p* = 0.062, ${\text{η}}_{p}^{2}$ = 0.10, with the VR condition reporting higher levels of control.

Furthermore, the level of reported negative mood after the trauma induction (mood ratings 2) predicted the number of intrusions, β = 0.39, *p* < 0.05, *R*^2^ = 0.15, and the reported level of distress, β = 0.52, *p* = 0.001, *R*^2^ = 0.25, *F*s > 6.50, *p*s < 0.015, indicating that a more negative mood resulted in more frequent and more distressing intrusions^6^. No other significant models emerged, *F*s < 1.

### Risk Factors and Intrusions

As previous analyses indicated that the intrusion characteristics were differently affected by type of trauma induction, we decided to separately analyze the data of the TFP and VR condition.

#### TFP Condition

Regression analyses (stepwise) were carried out with the total QMI score, BDI-II-NL, STAI-DY as independent variables and intrusion frequency and intrusion characteristics as dependent variables. These analyses resulted in one significant model, *F*(1, 17) = 4.52, *p* < 0.05, with the QMI being a predictor for the amount of distress of the intrusions, β = 0.46, *p* < 0.05, *R*^2^ = 0.16; that is, lower imagery ability predicted more distress of the intrusions.

#### VR Condition

Similar regressions were carried out for the VR condition. For the VR condition a significant model emerged for the number of intrusions, *F*(1, 18) = 11.56, *p* < 0.005. The QMI was the only significant predictor, β = –0.63, *p* < 0.005, *R*^2^ = 0.36, with more imagery abilities corresponding with more intrusions.

## Discussion

The aims of the present study were to consider the possibility of using VR as a novel analogue for PTSD and to assess PTSD risk factors. To this end a mild trauma was induced in healthy participants by presenting them either an aversive film (TFP) of physical abuse or a VR scene with a similar content. Risk factors that were assessed were trait anxiety, depression and imagery ability. The week following the trauma induction the frequency and characteristics of the intrusions were recorded using an Intrusion diary (cf. Brewin and Saunders, 2001; Holmes et al., 2004; Hagenaars and Arntz, 2012).

Concerning the first aim, the VR paradigm did induce a negative mood and involuntary intrusions in the week following the trauma induction. This change in negative mood and number of intrusions was comparable to that induced by the TFP. However, the persons in the TFP reported more distress of the intrusions and a trend was observed for less control compared to the VR condition. This suggests that the TFP seemed to be more effective for inducing PTSD-like symptoms. Regarding immersion, VR did result in higher levels of feeling present at the scene and more engagement. This is important as this feeling of “presence” is related to the level of emotions, such as anxiety (Riva et al., 2007), with stronger immersion leading to more intense emotions (Visch et al., 2010). As increases in negative mood are related to intrusion frequency (Davis and Clark, 1998), also indicated in the present study, VR seems to be a promising technique for inducing an analogue trauma.

Regarding the second aim, the risk factors, for the TFP lower imagery ability predicted more self-reported distress of the intrusions. In the VR condition higher imagery abilities predicted more intrusions; no predictive effects of the other risk factors, high trait anxiety and depression, were observed. These results partially confirm our hypotheses; higher imagery abilities coincided with more intrusions in the VR condition, but contrary to our expectations, no predictive effects of trait anxiety and depression were detected.

As the present study is the first to use VR for trauma induction, no direct comparisons concerning previous research can be made. The results of the TFP are in line with previous studies in that aversive film scenes are able to induce a negative mood and film-content related intrusions (Holmes and Bourne, 2008; Weidmann et al., 2009). Likewise, the VR paradigm resulted in a negative mood and induction-related intrusions. In accordance with previous studies, VR resulted in feeling present at the scene (Riva et al., 2007) and more immersion compared to a (3D) movie (Visch et al., 2010). Furthermore, like the study of Slater et al. (2013) emotions elicited were in line with the scene displayed, that is participants reported a higher negative mood after the VR induction (e.g., more anger, more sullenness, and disgust). However, unlike our expectations VR did not result in stronger changes in negative mood or more (distressful) intrusions compared to the TFP. These results can easily be explained by the general experimental set-up. As the present study was the first to induce a trauma with VR, a better safe than sorry strategy was adopted to protect the participants. That is, the VR scenes were quite mild compared to the TFP scenes. Though the content was similar, less violence and less visible physical abuse was used to level out distress increments due VR immersion. As the results indicate, the TFP resulted in more distressing intrusions, indicating that this paradigm seemed to be more aversive. Furthermore, the VR paradigm was made by a small team of engineers and scientists (a total of 5 persons) with a limited budget (€1.000); whereas for the film a large production team (over 400 persons) and budget ($13.000.000) was available. To make a fair comparison between film and VR, the VR scenes should be compared to a 2D film version of the same scenes. For future studies, it would be highly interesting to make such comparison and test the trauma induction qualities of both versions.

Concerning the risk factors, these results partly deviate from previous studies (Laposa and Alden, 2008). The BDI and STAI-DY scores were not predictive of the number or characteristics of the intrusions. A possible explanation is that including the QMI in the analyses might have obscured the effects of the BDI and STAI-DY. However, rerunning the analyses without the QMI did not yield any significant model for the BDI and STAI-DY. Note that the lack of a direct relation between intrusions and trait anxiety or depressive symptoms in an analogue trauma is not uncommon (Davis and Clark, 1998; Morina et al., 2013). A possible explanation is the difference in range and variation between the BDI, STAI-DY and QMI scores. For the BDI half of the participants scored a 0, indicating no depressive symptoms at all and persons with a score over 20 were excluded. The percentage of the maximum range (observed ranged divided by maximum range) for the BDI and STAI was 25 and 39%, respectively, indicating little dispersion of scores. This range was much larger for the QMI, 57%, leaving more variation and possibilities to detect predictive effects for the QMI on the outcome measures.

Regarding imagery ability, the analyses indicated that for the film low imagery ability predicted more distress of the intrusions. For VR higher imagery abilities coincided with more intrusions. The latter observation is conform Morina et al. (2013), with higher abilities resulting in more intrusions (VR condition), but unlike Morina et al. (2013) higher abilities related to less distress of the intrusions (TFP condition). This is a rather counterintuitive observation. A possible explanation is that persons with higher levels of imagery ability more often experience intrusions and that these intrusions can function as imaginal exposure, resulting in a reduction in distress of the involuntary memories (see for a clinical study about imaginal exposure Arntz et al., 2007). An alternative explanation is that persons with higher imagery abilities use (unconsciously) different coping strategies when confronted with unwanted intrusive memories, such as conceptual processing (Kindt et al., 2007) or memory elaboration (Ehlers et al., 2010). Though this might have been the case, no questionnaires or interview regarding such strategies were conducted in the present study.

A discrepancy was observed between the TFP and VR paradigm. Only in the latter higher imagery abilities coincided with more intrusions. This observation can be explained by differences in the amount of details and visibility of the physical injuries. The TFP contained close up daylight scenes with a high level of detail, leaving little space for imagination. The VR scene took place at night in a dark alley that was illuminated by streetlights. It is well thinkable that only in case of the VR paradigm imagery ability played an important role as this scene was open for visualization of non-displayed images.

A noteworthy observation is that VR has been successfully applied in exposure interventions for patients with PTSD symptoms (Gonçalves et al., 2012). However, VRET was used to reduce symptoms and not, as in the present study, to induce symptoms. This is an important difference as in our study we did not try to mimic a previously-experienced traumatic event, but tried to create a novel traumatic experience. It might be harder to establish a traumatic memory using VR than to retrieve or trigger a consolidated aversive memory using VR. Though the aim of the present study was not examine the efficacy of different PTSD interventions, the general experimental set up does allow such a comparison. That is, using VR as trauma induction guarantees control over the trauma onset, duration and type of traumatic event. This makes it easier to, subsequently, compare different PTSD treatments as patients do vary on these trauma-related aspects. Additionally, VR allows testing the efficacy of each intervention on avoidance of trauma-related material by manipulating VRE. The other way around can we test the influence of exposure in multiple settings, thereby promoting generalization, on future avoidance behavior. This is highly important as avoidance is one of the core symptoms of PTSD (Brewin et al., 2009).

The present study has a number of limitations. First, the VR trauma induction was short and quite mild to ensure that no long-lasting PTSD-symptoms would be evoked. As a result the intrusion frequency and distress levels were quite low. Second, the number of participants in each condition was quite small. This was done as the present study was a first set-up for the development of a novel PTSD analogue. Furthermore, a non-clinical population was used, making it difficult to generalize the results to a clinical population. Finally, we did not preselect our participants resulting in a limited range of variation in the questionnaires linked to the risk factors. For future research we would recommend to use a longer and more distressing VR scene, to preselect participants (e.g., high and low trait anxiety, imagery ability or depressive symptoms) and to measure putative coping strategies after trauma induction.

In conclusion, the current study is the first that uses VR as a tool for trauma induction. The results indicate that a VR paradigm can result in PTSD-like symptoms as negative mood and induction-related intrusions. Most importantly, using VR resulted in higher levels of immersion compared to the traditional film material. In sum, VR is a promising and feasible technology for trauma-related research.

### Conflict of Interest Statement

The authors declare that the research was conducted in the absence of any commercial or financial relationships that could be construed as a potential conflict of interest.

## Footnotes

1 http://www.phasespace.com/

2 http://www.worldviz.com/

3 http://www.blender.org/

4 http://www.autodesk.com/

5 http://www.richardbenning.nl/

6 Note that including gender as a predictor in the regression analyses carried out in Section “Intrusions” and “Risk Factors and Intrusions” yielded the same results.
